# Socioeconomic inequality in depression and anxiety and its determinants in Iranian older adults

**DOI:** 10.1186/s12888-022-04433-w

**Published:** 2022-12-05

**Authors:** Zahra Azizabadi, Nayyereh Aminisani, Mohammad Hassan Emamian

**Affiliations:** 1 Student Research Committee, School of Public Health, Shahroud University of Medical Sciences, Shahroud, Iran; 2 Healthy Ageing Research Centre, Neyshabur University of Medical Sciences, Neyshabur, Iran; 3 Ophthalmic Epidemiology Research Center, Shahroud University of Medical Sciences, Shahroud, Iran

**Keywords:** Inequality, Socioeconomic status, Concentration index, Depression, Anxiety

## Abstract

**Background:**

Older adults with lower socioeconomic status are more vulnerable to stressful life events and at increased risk of common mental health disorders like anxiety and depression. This study investigates the socioeconomic inequality in depressive symptoms and anxiety.

**Methods:**

The data were from 7462 participants of the Neyshabur longitudinal study of ageing registered during 2016-2018. The outcome variables were anxiety and depressive symptoms. Anxiety was defined by the “Hospital Anxiety and Depression scale Questionnaire”, and depressive symptoms was defined and measured by the “short-term form of the Epidemiological Center Questionnaire.” The socioeconomic status was defined using principal component analysis of home assets. The Concentration Index (C) was used to measure socioeconomic inequality in anxiety and depressive symptoms. Concentration index was decomposed to its determinants to determine the role of the independent variables on inequality.

**Results:**

The prevalence of depressive symptoms and anxiety was 12.2% (95% CI: 11.4, 12.9) and 7.0% (95% CI: 6.4, 7.5), respectively. Moreover, the C for anxiety was -0.195 (95% CI: -0.254, -0.136) and for depressive symptoms was -0.206 (95% CI: -0.252, -0.159), which indicate a considerable inequality in favor of high socioeconomic group for anxiety and depressive symptoms. Decomposition of the concentration Index showed that education, unemployment and male sex were the most important positive contributors to the observed inequality in anxiety and depressive symptoms, while age and number of grandchildren were main negative contributors of this inequality.

**Conclusion:**

Low socioeconomic groups were more affected by anxiety and depressive symptoms. Any intervention for alleviation of inequality in anxiety and depression should be focus on education and employment of people, especially in younger elderly.

## Background

Over the last decades, the number of older people has increased significantly from 130 million in 1950 to more than 600 million in 2017 [1]. It has been predicted that from 2015 to 2050, the ratio of people >60 will almost double, from 12% to 22%, globally [2]. In Iran, according to the population censuses, the elderly accounted for 6.69% of the total population and it has been estimated that population aged 65 years or over will increase to 18.25% by 2050 [3].

Depression is defined as persistent sadness and lack of interest and pleasure in doing formerly delightful activities [4]. According to the World Health Organization, 279 million people, or 3.7% of the world's population, suffer from depression [5]. In Iran, the prevalence of depression equals 5.4% in 2019 [5], with a Years of healthy life lost due to disability (YLD) equal to 813,441 years (8.5% of total YLD) [5]. The prevalence is associated with age and it was reported up to 52% in elderly [6]. According to the World Health Organization statistics, 301 million people, or 4.0% of the world's population are experiencing anxiety throughout their lives. In Iran, the overall prevalence of anxiety was 7.8% in 2019, and YLD for anxiety was 608,056 years (6.4% of total YLD) [7].

Inequality in health is defined as the discrepancy in the incidence or prevalence of health problems among individuals in different situations (economic, social, geographical, etc.) [8]. Low-income countries usually have more inappropriate health outcomes than wealthy countries. Moreover, in all the countries worldwide, the lower socioeconomic groups suffer, the more disease burden than the higher classes [9, 10]. A healthy society program aims to eliminate health inequalities among the genders, with different ethnicities, races, educational status, income levels and geographical locations [11]. Therefore, one of the principal goals of global public health is striving against social and economic inequalities. The World Health Organization recommends monitoring and evaluating socioeconomic inequalities in health behaviors as one of the social determinants of health [10]. There are pieces of evidence that socioeconomic inequality of depression and anxiety directly correlates with age [12, 13]. A meta-analysis study among adults found that the lower the socioeconomic status was associated with higher prevalence or incidence of depression (a pro-rich inequality) [14]. The inequality in health status is avoidable in many cases through adjustable factors such as economic status, education status, employment, and living facilities [15]. A few studies have been done in Iran on the effects of socioeconomic inequality in mental health, which illustrates that this inequality is often in favor of the rich and has a relation with features such as gender, age, and employment status [16–19].

The current study aims to determine socioeconomic inequality in depressive symptoms and anxiety and characterize the determinants of these inequalities based on a large population-based study in Northeast of Iran; Nayshabur Longitudinal Study on Ageing (NeLSA). Identifying the status of socioeconomic inequality in depression and anxiety and its determinants will help policymakers to implement appropriate interventions and promote mental health in society.

## Methods

### Study population

The data was extracted from the Neyshabour longitudinal study on aging (NeLSA) [20], which is an ageing component of the Prospective Epidemiological Research Studies in Iran (PERSIAN) [21]. It was conducted in four sites, including Neyshabur (Razavi Khorasan province, Northeast of Iran), Guilan (Northern Iran), Tabriz (Northwest of Iran), and Ardakan (central Iran). The current study included people aged 50 -94 in Neyshabur during 2016-2018. Participants were selected through stratified random sampling from people registered with six health centers. A total of 9220 people met the eligibility criteria including minimum 3-year residency in Neyshabur, Iranian citizens, without dementia, major depression, and disabilities, limiting their ability to participate in the study, of whom a total of 7462 individuals (4831 households) provided the written consent to participate in the study. The participation rate was 81%. Details of NeLSA sampling and implementation have already been reported [20].

### Independent and Outcome variables

The outcome variables of the current study were depressive symptoms and anxiety. The "Short-Form of the Center for Epidemiological Studies–Depression Scale (CES-D)" [22] and "The Hospital Anxiety and Depression Scale" [23] were used respectively to assess depressive symptoms and anxiety. A score of 11 or higher was considered as the anxiety disorder and the score of 10 or higher was considered positive for depressive symptoms. Mentioned questionnaires are considered effective screening tools due to their good reliability, validity, and sensitivity, based on the results of the former studies [23–25]. Both 10 and 8 items forms of CES-D were also validated for Farsi language and Iranian elderly [25].

Trained officers conducted face-to-face interviews using a comprehensive questionnaire. It includes information related to sociodemographic (age, sex, marital status, education, income, and job), lifestyle behaviors (smoking, physical activity, diet, sleep), history of chronic disease, and medication use. Trained clinical psychologists completed psychological questionnaires (cognition, quality of life, depression, anxiety, etc.). There was a clinical examination by a physician or trained nurse. All procedures were based on standard protocols followed by a quality control check.

Chronic diseases were defined by a physician on clinical assessment and the participant’s response to the question ‘Has a doctor ever told you that you have any of the following health problems? In this study, a list of different chronic diseases, including gastrointestinal, cardiac, neurologic, musculoskeletal, endocrine, respiratory, and cancers, have been investigated. Participants had been asked to bring all medical records, laboratory results, and medications that they were using on the interview day; they were all checked by a general practitioner to verify the self-reported medical conditions.

Diabetes was defined as self-report history of diabetes and/or using diabetes medications and/or FBS>=126 in a blood test. Hypertension was defined as self-report history of hypertension and/or using hypertension medications and/or systolic blood pressure >=140 mmHg and/or diastolic blood pressure >=90 mmHg. Smoking behavior was based on whether respondents identified themselves as a regular smoker or not.

Body Mass Index was calculated after measuring weight and height of participants and was categorized as normal (< 25 Kg/m^2^), overweight (25–29.9 Kg/m^2^) and obese (≥30 Kg/m^2^). Marital status was classified into two groups: married/living with a partner and divorced/separated/single/widow.

### Socioeconomic status variable

Principal Component Analysis was used to construct a variable that shows the socioeconomic status [26–28]. Several factors were considered in the PCA model to generate a socioeconomic status variable. It included the Possession of a freezer, washing machine, dishwasher, laptop / desktop, Internet access, LCD / LED TV, vacuum cleaner, master bedroom (built-in bathroom in the bedroom), motorcycle, the car value 5000-12500$, tablet / IPAD. The number of extracurricular and non-professional books read in the past year and the number of foreign and domestic trips in the last ten years entered into the model as a social status variable. Categorical variables were re-coded as binary variables (0 and 1), then all continuous and binary variables were entered into the model. As a result, seven components were obtained with Eigenvalue> 1, covering 61.99% of the observed variance. The Sum of the asset variables weighted by the first component was used to calculate the socioeconomic score for each individual [26].

### Inequality measurement

The Concentration Index (C) was measured to evaluate inequality, which has been widely used to examine income-related inequalities in the health departments internationally. Its decomposition analysis is increasingly being used to study the determinants of health inequality in elderly [29, 30].

To understand concentration index, one must first become familiar with the concentration curve, which in the present study displays the share of depressing or anxiety accounted for by cumulative proportions of individuals in the population ranked from lowest to highest socioeconomic status. The x-axis of concentration curve demonstrates the cumulative percentage of population, ranked by their socioeconomic status and the y-axis presents the cumulative percentage of health outcome (depressive symptoms or anxiety). Therefore, if individuals - regardless of their economic status - have equal health outcomes, the curve will be a line of 45 degrees (equality line) [31].

The concentration index is defined as twice the area between the concentration curve and the line of equality. The C value becomes negative when the outcome under study is concentrated among the lower socioeconomic groups. In this scenario the concentration curve will be above the equality line. On the contrary, the value of the C becomes positive when the concentration curve is below the equality line, and the outcome under study is concentrated among the higher socioeconomic groups. Hence, the higher the absolute value of the index, the greater the inequality. The C ranges from +1 (the outcome under study is entirely focused on the rich) to -1 (the outcome under study is entirely focused on the poor), and a value of zero indicates equality [31, 32]. The concentration index was calculated using the “conindex” command [33] with the option for bounded outcome variables, in Stata 15 software (College Station, TX: StataCorp LLC). Participants with complete data in all above parts were included in this study.

The Wagstaff decomposition method was used in the current study [34]. The C has two components: the explained component that identifies each determinant's contribution to socioeconomic inequality, and the unexplained or residual component (derived from βs), specifies which socioeconomic inequality is not explained through the systematic variation of the determinants among socioeconomic groups. Elasticity is a measure of the association without unity; it shows the importance of the variation of the dependent variable per unit of change in the determinant. To calculate elasticity; the beta coefficient of any independent variable was multiplied by the mean of the same variable. The result was divided by the mean of the outcome variable. For each determinant variable, the multiplication of the elasticity and concentration index indicates the absolute contribution of that determinant. Moreover, to stipulate the percentage contribution of each determinant, the absolute contribution is divided by the concentration index of the dependent variable [34]. Given the binary outcomes in this study, we used linear approximation by using marginal effects on the logit model, as coefficients.

## Results

The data of 7462 participants were used for the current analysis. The mean and standard deviation of the age of the participants equals 61.0±8.14 years, and most of them had primary education. Most of the participants (90%) lived with an individual or persons. The prevalence of overweight and obesity was 43.0% and 29.9% respectively. Smokers accounted for 10.9% of the population.

The data of 7462 and 7316 participants were available for depressive symptoms and anxiety scores. The mean and standard deviation (SD) of test scores for depressive symptoms and anxiety disorders were 3.94 (4.16) and 5.15 (3.28), respectively. The prevalence of depressive symptoms and anxiety in total population was 12.2% (95% CI: 11.4 – 12.9) and 7.0% (95% CI: 6.4 – 7.6) respectively. More information on how these two disorders were distributed to other independent variables is appointed in Table 1. The prevalence of depressive symptoms and anxiety were different in socioeconomic groups. Within the highest SES group; depressive symptoms, and anxiety were 6.5% and 3.4%, while within the lowest SES group, they were equal to 16.2% and 9.7%, respectively. The prevalence of depressive symptoms was not different in different age groups (*p*=0.287), while anxiety was less frequent in higher age groups (*p*=0.003)

**Table 1:** Distribution of anxiety and depressive symptoms by demographic and prior medical history, Neyshabour, Iran, 2016-2018

Variables		N (%)	Depressive symptoms		Anxiety	
			N (%)	*P* Value	N (%)	*P* Value
Age groups	50 - 59	4010 (53.7)	508 (12.7)	0.287	313 (8.0)	0.003
	60 - 69	2338 (31.3)	260 (11.1)		146 (6.4)	
	70 - 70	877 (11.8)	112 (12.8)		43 (5.0)	
	≥ 80	237 (3.2)	27 (11.4)		11 (4.7)	
Sex	Female	3962 (53.1)	609 (15.4)	<0.001	384 (9.9)	<0.001
	Male	3500 (46.9)	298 (8.5)		129 (3.8)	
Smoking	Yes	760 (10.9)	99 (13.0)	0.170	45 (6.0)	0.237
	No	6189 (89.1)	702 (11.3)		436 (7.1)	
	No	3825 (56.9)	408 (10.7)		227 (6.0)	
Chronic disease	Yes	5451 (73.1)	704 (12.9)	0.001	417 (7.8)	<0.001
	No	2011 (26.9)	203 (10.1)		96 (4.9)	
Socioeconomic quintiles	1 (Lowest)	1169 (20.1)	189 (16.2)	<0.001	112 (9.7)	<0.001
	2	1167 (20.0)	163 (14.0)		88 (7.7)	
	3	1169 (20.0)	128 (11.0)		87 (7.6)	
	4	1167 (20.0)	110 (9.4)		68 (5.9)	
	5 (Highest)	1168 (20.0)	76 (6.5)		39 (3.4)	
Body Mass Index	Underweight	130 (1.8)	19 (14.6)	0.002	11 (8.6)	0.305
	Normal	1822 (25.2)	224 (12.3)		122 (6.8)	
	Overweight	3105 (43.0)	312 (10.1)		201 (6.5)	
	Obese	2160 (29.9)	287 (13.3)		166 (7.8)	
Occupation status	employed	6808 (92.4)	777 (11.4)	<0.001	463 (6.9)	0.130
	unemployed	563 (7.6)	80 (14.2)		48 (8.6)	
Education	illiterate	1934 (26.3)	326 (16.9)	<0.001	189 (9.9)	<0.001
	Primary	2514 (34.1)	299 (11.9)		176 (7.1)	
	Secondary	666 (9.0)	74 (11.1)		46 (7.0)	
	Diploma	1527 (20.7)	139 (9.1)		81 (5.4)	
	University	723 (9.8)	22 (3.0)		18 (2.5)	

The concentration indices for anxiety and depressive symptoms were -0.195 (95% CI: -0.254, -0.136) and -0.206 (95% CI: -0.252, -0.159), respectively. The negative concentration index implies that inequality was favored to the high SES group, and anxiety and depressive symptoms were concentrated in the low SES group.

**Fig. 1:**
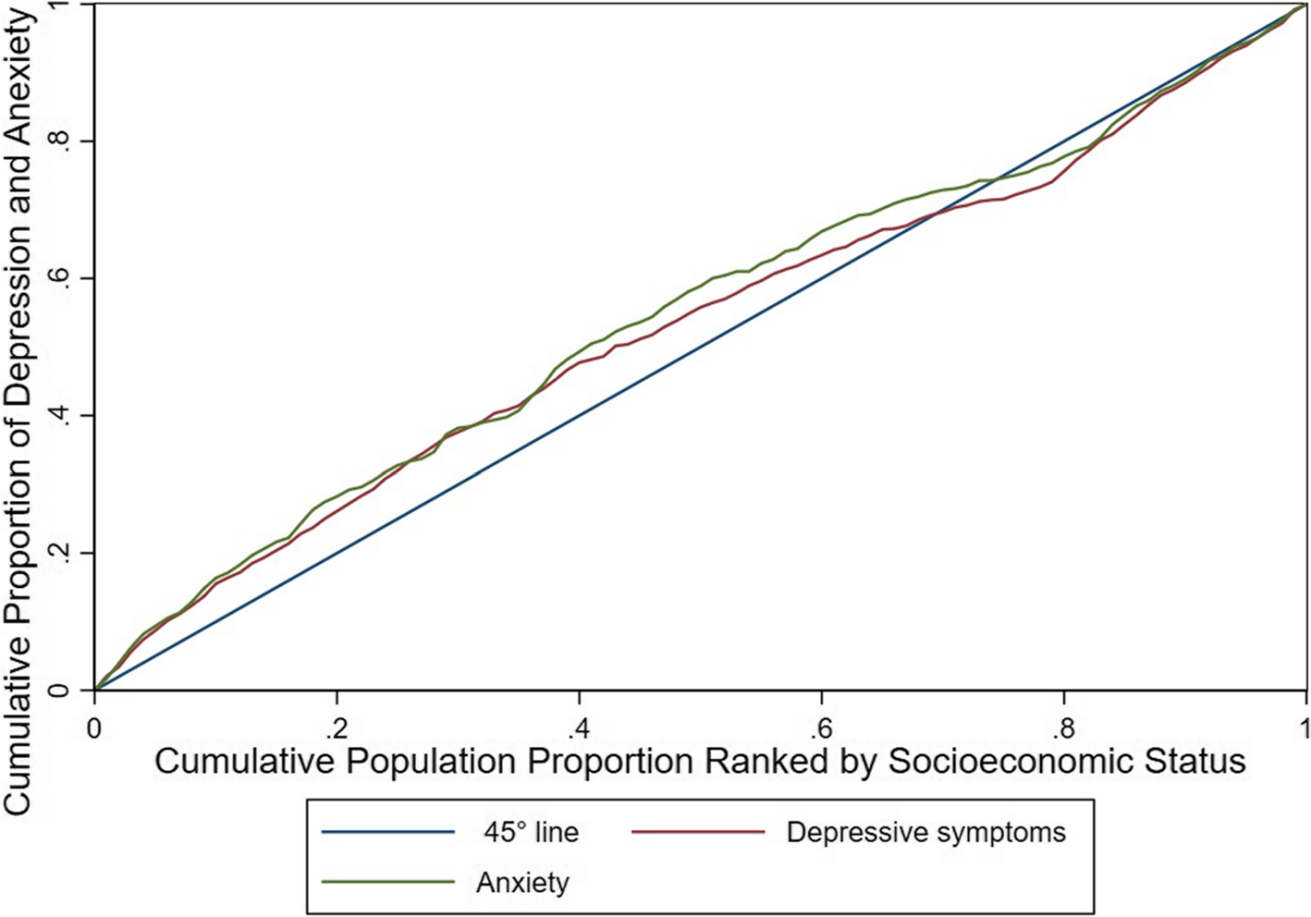
Concentration curve for depressive symptoms (red line) and anxiety (green line) by socioeconomic status Neyshabour, Iran, 2016-2018

Figure 1 illustrates the concentration curve of anxiety and depressive symptoms. The concentration curves for both disorders were above the equality line, indicating a pro-rich inequality.

Table 2 represents the decomposition of the concentration index for anxiety. Among the studied variables, education, age, sex, occupational status, and number of grandchildren were the common determinants of inequality. The large elasticity of anxiety with respect to age is responsible for its large contribution to the anxiety concentration index. In contrast, there is a great deal of socioeconomic inequality in the numbers of grandchildren, education and age, and so they make large contribution to the anxiety concentration index. Education and age make the largest proportional contributions to overall socioeconomic inequality.

**Table 2:** Results for the decomposition of the concentration index for anxiety in Neyshabour, Iran, 2016-2018

Independent Variables	Βeta^*^	Mean	Elasticity	Concentration Index (C)	Absolute Contribution	% Contribution to C
Gender (male)	-0.068	0.469	-0.453	0.137	-0.062	31.9
Age (year)	-0.002	61.083	-1.837	-0.058	0.107	-55.1
BMI	-0.001	27.815	-0.554	0.010	-0.006	3.0
Smoking	0.020	0.109	0.031	-0.044	-0.001	0.7
Chronic disease	0.023	0.730	0.239	0.007	0.002	-0.8
Grandchildren (No.)	-0.001	5.430	-0.080	-0.267	0.021	-10.9
Great grandchildren (No.)	0.002	0.373	0.012	-0.515	-0.006	3.3
Education (Year)	-0.004	5.519	-0.332	0.427	-0.142	72.6
Living alone	0.017	0.101	0.024	-0.378	-0.009	4.8
Unemployment	0.061	0.076	0.025	-0.351	-0.023	12.0

^*^Marginal effects on logit model

Similar pattern for the contribution of variables in depressive symptoms inequality was also seen (Table 3), where education, age and sex had the highest contributions to depressive symptoms inequality.

**Table 3:** Results for the decomposition of the concentration index for depressive symptoms in Neyshabour, Iran, 2016-2018

Independent Variables	Βeta^*^	Mean	Elasticity	Concentration Index (C)	Absolute Contribution	% Contribution to C
Gender (male)	-0.072	0.469	-0.278	0.137	-0.038	18.6
Age (year)	-0.001	61.083	-0.690	-0.058	0.040	-19.6
BMI	-0.001	27.815	-0.332	0.010	-0.003	1.7
Smoking	0.057	0.109	0.051	-0.044	-0.002	1.1
Chronic disease	0.046	0.730	0.279	0.007	0.002	-0.9
Grandchildren (No.)	-0.002	5.430	-0.081	-0.267	0.022	-10.6
Great grandchildren (No.)	0.003	0.373	0.009	-0.515	-0.005	2.3
Education (Year)	-0.007	5.519	-0.334	0.427	-0.142	69.3
Living alone	0.030	0.101	0.025	-0.378	-0.010	4.7
Unemployment	0.056	0.076	0.035	-0.351	-0.012	6.0

^*^Marginal effects on logit model

## Discussion

In this study, the concentration index of anxiety and depressive symptoms to determine socioeconomic inequality were equal to -0.195 and -0.206, respectively, which has been indicated a significant inequality in anxiety and depressive symptoms. Similar to other studies [35, 36], the inequality in anxiety was concentrated among individuals with low socioeconomic status. The prevalence of this disorder was 9.7% within the lowest and 3.4% within the highest SES quintiles. The prevalence of depressive symptoms equals 16.2% in the lowest and 53.46.5% in the highest SES quintiles. Although depression has been as often as possible conceptualised as a ‘backward-looking’ emotion, and anxiety as ‘forward-looking’ [37, 38], both were associated with socioeconomic status in current study. The prevalence of depression, which can affect inequality is very different across nations [39–41]. Contrary to our results another study in three European countries [42] find no association between income and depression in Spain, while in Finland and Poland with lower prevalence of depression, a pro-rich inequality in depression was reported. The above comparison between studies indicates that the socioeconomic inequality in depression is heterogeneous and other factors including the way of measurement and definition of depression and SES, the region, the study time, study population and its sample size should be considered [14]. In a study in Finland, Poland and Spain [43], higher income was associated with lower odds of depression in a logistic regression model adjusted for age and sex. This association was not significant in another model with adjustment for age, sex and other demographic, behavioral variables and chronic diseases. Therefore, the analysis method and plan, is another reason for differences between studies. Similar to our results, other studies in Spain [44] Korea [45], India [46], South Africa [47], and US [48], have shown a pro-rich inequality in depression with different extents. Richardson’s study [49] focuses on the potential role that the social environment within countries may play in shaping inequalities and differences between countries.

We found a socioeconomic inequality in anxiety which was in favor to high socioeconomic groups. This finding is in accordance with other studies around the world [50–53].

Decomposition of inequality in anxiety and depressive symptoms expressed that the main modifiable factors, causing this inequality in terms of C were: education level, number of grandchildren (negative contribution) and employment status. According to the different values of elasticity and concentration index in each studied variable, the contribution percentages of factors affecting inequality are different. So that age and education played most significant role in the inequality of anxiety and depressive symptoms.

Current study represents that, elderly with low education will experience anxiety and depressive symptoms more. This finding has been reported in many studies, especially in developing countries, which reveals that lower levels of education were significantly associated with mental disorders such as anxiety, depression, and stress [54, 55]. Education was the factor that contributed the most to the socioeconomic inequality of anxiety and depressive symptoms in the decomposition of the concentration index. About 70% of the inequality observed in anxiety and depressive symptoms was explained by the education level. This result was consistent with the findings of other studies [56, 57]. This could be explained by the fact that individuals with higher levels of education are more aware of prevention ways of anxiety, which improves their mental status [58]. The Low level of education is an independent risk factor for anxiety; thus, interventions in education at the community level can be considered a way to reduce socioeconomic inequalities of anxiety [59]. Education is one of the social factors affecting health, which can create a strong network of communication and more social links for the elderly, and successively will cause a better mental health state [59]. Higher education levels also help adults in prevention of diseases, health promotion, access to health insurance, and having healthy behaviors [60]. It seems that literacy is a development index in older people, as the second leading indicator of vulnerability of mental status. Consequently, adopting policies aimed to increase literacy among individuals with lower socioeconomic status could be one of the most substantial steps in reducing socioeconomic inequality of anxiety.

Age was the second most contributor to inequality of anxiety and depressive symptoms. The negative contribution of age with depressive symptoms and anxiety inequality, means that increasing in age associated with lower inequality and people in older age affected more equal with depressive symptoms and anxiety. Therefore, any intervention to reduce the socioeconomic inequality in anxiety and depressive symptoms of the elderly (such as literacy increasing interventions) should be more focused on younger age groups. Other studies have also reported the role of age in socio-economic inequality in mental health, some of them have reported that anxiety, depressive symptoms and mental health worsen with age [18, 19, 40]. The reason for this discrepancy is the difference in the age ranges of this study and other studies. Most other studies have been in adults of all age groups [19, 61]. Of course, some studies in old age also had similar results to ours [46, 61].

Not having an occupation was another contributor to anxiety and depressive symptoms inequality. It seems that having even a part-time job can play a key role in reducing anxiety [62]. Therefore, attention to employment status in elderly is important to minimize inequality in depressive symptoms and anxiety.

Further analysis of data in this study showed that 21.24% of lonely and more anxious elderly were within the low socioeconomic groups while 5.12% were in the high socioeconomic groups. Other studies have shown that living alone was strongly associated with depressive symptoms and depression [57, 63–65] and anxiety [66] in elderly. In this regard, Berkman believes that social support creates a sense of intimacy through emotional patronage. He daresay family is the most remarkable factor in the loving communication establishment or emotional support [67]. Other study in India also indicates the protective role of a good social network against depression, in elderly [68].

The United Nations, in its 17 Sustainable Development Goals, has emphasized the necessity of paying more attention to the factors impacting economic and social conditions on health inequalities, including education, policy decisions engagement, employment, and socioeconomic differences. Although the principal causes of inequality in anxiety and mental disorders may vary by region, culture, and gender, efforts should be taken place to address them [69]. It should be also noted that other country level factors that were not investigated in this study, also have an effect on depression and anxiety [70].

High sample size, good design, robust analysis of data, proper implementation of study and systematic monitoring of study to ensure quality assurance in data gathering are the main strength of this study. However, exclusion of patients with major depression limits comparability of our results with some other studies and to some extent underestimate the inequality. Limited age range of participants to over 50 years, also Limits the generalizability of the results. As another limitation, it should be noted that decomposition of inequality has been done based on the factors that were examined in the questionnaire. Evidently, all effective factors were not examined in the present study. finally, no causal inference can be drawn from this cross-sectional study. What was described as a reason for inequality of anxiety is just the association between the variables under study, and there is no causal role. Therefore, it is recommended that this methodology be used to study inequalities in longitudinal studies with an appropriate design.

## Conclusion

Lower socioeconomic groups were more affected by anxiety and depressive symptoms among older adults of Neyshabur. Lower education, unemployment, and younger age were the main factors that play a considerable role in the inequality of anxiety and depressive symptoms. These factors should be considered for policymaking and for the development of new interventions to lower prevalence of anxiety and depressive symptoms in elderly.

## Data Availability

The datasets analyzed during the current study are available from the corresponding author on reasonable request.

## Abbreviations

PERSIAN: Prospective epidemiological research in Iran
SES: Socioeconomic status
BMI: Body mass index
C: Concentration index
NeLSA: Neyshabour longitudinal study on aging
YLD: Years of healthy life lost due to disability
CI: Confidence interval
PCA: Principal Component Analysis
SD: Standard Deviation

## References

[1] Ogura S, Jakovljevic MM (2018). Global population aging-health care, social and economic consequences. Front Public Health.

[2] World Health Organization (2021). Blindness and vision impairment.

[3] Statistical Center of Iran. Investigating the trend of changes in the structure and composition of the country’s population and its future: Statistical Center of Iran; 2019. Available from: https://www.amar.org.ir/Portals/0/Files/fulltext/1398/N_brtsvtjkvaato1430.pdf.

[4] World Health Organization (2020). Depression-Health topics.

[5] Institute of Health Metrics and Evaluation GHDE, (GHDx). 2019. Available from: http://ghdx.healthdata.org/gbd-results-tool?params=gbd-api-2019-permalink/d780dffbe8a381b25e1416884959e88b.

[6] Jafari H, Ghasemi-Semeskandeh D, Goudarzian AH, Heidari T, Jafari-Koulaee A (2021). Depression in the Iranian elderly: a systematic review and meta-analysis. J Aging Res.

[7] World Health Organization (2017). Depression and other common mental disorders.

[8] Currie C, Zanotti C, Morgan A, Currie D, De Looze M, Roberts C, et al. Social determinants of health and well-being among young people. Health behaviour in school-aged children (HBSC) study: international report from the 2009/2010 survey. WHO Regional Office for Europe: WHO; 2009/2010.

[9] Smith MJ (2015). Health equity in public health: clarifying our commitment. Public Health Ethics.

[10] Marmot M, Friel S, Bell R, Houweling TA, Taylor S, CoSDo H (2008). Closing the gap in a generation: health equity through action on the social determinants of health. Lancet.

[11] Keppel K, Pamuk E, Lynch J, Carter-Pokras O, Kim I, Mays V, et al. Methodological issues in measuring health disparities. National Center for Health Statistics. Vital Health Stat 2. 2005;(141):1–16. https://stacks.cdc.gov/view/cdc/6654. PMC3681823 16032956

[12] Green M, Benzeval M (2011). Ageing, social class and common mental disorders: longitudinal evidence from three cohorts in the West of Scotland. Psychol Med.

[13] Chandola T, Ferrie J, Sacker A, Marmot M (2007). Social inequalities in self reported health in early old age: follow-up of prospective cohort study. BMJ.

[14] Lorant V, Deliège D, Eaton W, Robert A, Philippot P, Ansseau M (2003). Socioeconomic inequalities in depression: a meta-analysis. Am J Epidemiol.

[15] Zaboli R, Malmoon Z, Seyedjavadi M, Seyedin H (2014). Developing a conceptual model of social determinants of health inequalities: a qualitative study. J Health Promot Manag.

[16] Najafi F, Pasdar Y, Karami Matin B, Rezaei S, Kazemi Karyani A, Soltani S (2020). Decomposing socioeconomic inequality in poor mental health among Iranian adult population: results from the PERSIAN cohort study. BMC Psychiatry.

[17] Harouni GG, Mahdavi MRV, Naghdi S, Armoon B, Fazaeli AA, Ghiasvand H (2018). Decomposing disparity in adult individual’s mental health in Tehran among lower and higher economic groups; an Oaxaca-Blinder analysis on urban HEART Survey-round 2. Afr Health Sci.

[18] Morasae EK, Forouzan AS, Majdzadeh R, Asadi-Lari M, Noorbala AA, Hosseinpoor AR (2012). Understanding determinants of socioeconomic inequality in mental health in Iran's capital, Tehran: a concentration index decomposition approach. Int J Equity Health.

[19] Veisani Y, Delpisheh A (2015). Decomposing of socioeconomic inequality in mental health: a cross-sectional study into female-headed households. J Res Health Sci.

[20] Aminisani N, Azimi-Nezhad M, Shamshirgaran SM, Mirhafez SR, Borji A, Poustchi H (2022). Cohort profile: the Iranian longitudinal study on ageing (IRLSA): the first comprehensive study on ageing in Iran. Int J Epidemiol.

[21] PERSIAN Elderly Cohort, 2014. Accessed Audust 2022. Available from: https://persiancohort.com/elderly-cohort-design-and-objectives/.

[22] Kimberlin CL, Pendergast JF, Berardo DH, McKenzie LC (1998). Issues related to using a short-form of the center for epidemiological studies–depression scale. Psychol Rep.

[23] Herrero M, Blanch J, Peri J, De Pablo J, Pintor L, Bulbena A (2003). A validation study of the hospital anxiety and depression scale (HADS) in a Spanish population. Gen Hosp Psychiatry.

[24] Kuijpers PM, Denollet J, Lousberg R, Wellens HJ, Crijns H, Honig A (2003). Validity of the Hospital anxiety and depression scale for use with patients with noncardiac chest pain. Psychosomatics.

[25] López-Alvarenga JC, Vázquez-Velázquez V, Arcila-Martínez D, Sierra-Ovando AE, González-Barranco J, Salín-Pascual RJ (2002). Accuracy and diagnostic utility of the hospital anxiety and depression scale (HAD) in a sample of obese Mexican patients. Rev Investig Clin.

[26] Vyas S, Kumaranayake L (2006). Constructing socio-economic status indices: how to use principal components analysis. Health Policy Plan.

[27] Houweling TAJ, Kunst AE, Mackenbach JP (2003). Measuring health inequality among children in developing countries: does the choice of the indicator of economic status matter?. Int J Equity Health.

[28] Howe LD, Hargreaves JR, Huttly SR (2008). Issues in the construction of wealth indices for the measurement of socio-economic position in low-income countries. Emerg Themes Epidemiol.

[29] Goli S, Singh L, Jain K, Pou LMA (2014). Socioeconomic determinants of health inequalities among the older population in India: a decomposition analysis. J Cross Cult Gerontol.

[30] Wang Z, Li X, Chen M (2015). Catastrophic health expenditures and its inequality in elderly households with chronic disease patients in China. Int J Equity Health.

[31] O'Donnell O, van Doorslaer E, Wagstaff A, Lindelow M (2008). Analyzing health equity using household survey data: a guide to techniques and their implementation.

[32] Oakes JM, Kaufman JS (2017). Methods in social epidemiology.

[33] O'Donnell O, O'Neill S, Van Ourti T, Walsh B (2016). Conindex: estimation of concentration indices. Stata J.

[34] Wagstaff A, Doorslaer vE, Watanabe N. (2001). On decomposing the causes of health sector inequalities with an application to malnutrition inequalities in.

[35] Wildman J (2003). Income related inequalities in mental health in Great Britain: analysing the causes of health inequality over time. J Health Econ.

[36] Mangalore R, Knapp M, Jenkins R (2007). Income-related inequality in mental health in Britain: the concentration index approach. Psychol Med.

[37] Wenze SJ, Gunthert KC, German RE (2012). Biases in affective forecasting and recall in individuals with depression and anxiety symptoms. Personal Soc Psychol Bull.

[38] Mlawer F, Hubbard JA, Bookhout MK, Moore CC (2021). Levels and instability of daily self-esteem in adolescents: relations to depressive and anxious symptoms. Res Child Adolesc Psychopathol.

[39] Simon GE, Goldberg DP, Von Korff M, ÜStÜN TB. (2002). Understanding cross-national differences in depression prevalence. Psychol Med.

[40] Lu J, Xu X, Huang Y, Li T, Ma C, Xu G (2021). Prevalence of depressive disorders and treatment in China: a cross-sectional epidemiological study. Lancet Psychiatry.

[41] Szklo M, Nieto FJ (2019). Epidemiology: Beyond the Basics.

[42] Freeman A, Tyrovolas S, Koyanagi A, Chatterji S, Leonardi M, Ayuso-Mateos JL (2016). The role of socio-economic status in depression: results from the COURAGE (aging survey in Europe). BMC Public Health.

[43] Domènech-Abella J, Mundó J, Leonardi M, Chatterji S, Tobiasz-Adamczyk B, Koskinen S (2018). The association between socioeconomic status and depression among older adults in Finland, Poland and Spain: a comparative cross-sectional study of distinct measures and pathways. J Affect Disord.

[44] Costa-Font J, Gil J (2008). Would socio-economic inequalities in depression fade away with income transfers?. J Happiness Stud.

[45] Hong J, Knapp M, McGuire A (2011). Income-related inequalities in the prevalence of depression and suicidal behaviour: a 10-year trend following economic crisis. World Psychiatry.

[46] Muhammad T, Skariah AE, Kumar M, Srivastava S (2022). Socioeconomic and health-related inequalities in major depressive symptoms among older adults: a Wagstaff’s decomposition analysis of data from the LASI baseline survey, 2017-2018. BMJ Open.

[47] Mutyambizi C, Booysen F, Stornes P, Eikemo TA (2019). Subjective social status and inequalities in depressive symptoms: a gender-specific decomposition analysis for South Africa. Int J Equity Health.

[48] Dev S, Kim D (2020). State-level income inequality and county-level social capital in relation to individual-level depression in middle-aged adults: a lagged multilevel study. Int J Environ Res Public Health.

[49] Richardson RA, Keyes KM, Medina JT, Calvo E (2020). Sociodemographic inequalities in depression among older adults: cross-sectional evidence from 18 countries. Lancet Psychiatry.

[50] Melita D, Willis GB, Rodríguez-Bailón R (2021). Economic inequality increases status anxiety through perceived contextual competitiveness. Front Psychol.

[51] Panagiotakos DB, Pitsavos C, Tsetsekou E, Chrysohoou C, Kinlaw M, Papageorgiou C (2007). Anxiety and socio-economic status among healthy adults: the ATTICA study. Epidemiol Psichiatria Soc.

[52] Mwinyi J, Pisanu C, Castelao E, Stringhini S, Preisig M, Schiöth HB (2017). Anxiety Disorders are Associated with Low Socioeconomic Status in Women but Not in Men. Womens Health Issues.

[53] Silvernale C, Kuo B, Staller K (2019). Lower socioeconomic status is associated with an increased prevalence of comorbid anxiety and depression among patients with irritable bowel syndrome: results from a multicenter cohort. Scand J Gastroenterol.

[54] Mumford DB, Saeed K, Ahmad I, Latif S, Mubbashar MH (2018). Stress and psychiatric disorder in rural Punjab: a community survey. Br J Psychiatry.

[55] McCall NT, Parks P, Smith K, Pope G, Griggs M (2002). The prevalence of major depression or dysthymia among aged Medicare Fee-for-Service beneficiaries. Int J Geriatr Psychiatry.

[56] Srivastava S, Purkayastha N, Chaurasia H, Muhammad T (2021). Socioeconomic inequality in psychological distress among older adults in India: a decomposition analysis. BMC Psychiatry.

[57] Aziz R, Steffens DC (2013). What are the causes of late-life depression?. Psychiatr Clin North Am.

[58] Ribeiro O, Teixeira L, Araújo L, Rodríguez-Blázquez C, Calderón-Larrañaga A, Forjaz MJ (2020). Anxiety, depression and quality of life in older adults: trajectories of influence across age. Int J Environ Res Public Health.

[59] Schiffman J, Reeves GM, Kline E, Medoff DR, Lucksted A, Hoagwood K (2015). Outcomes of a family peer education program for families of youth and adults with mental illness. Int J Ment Health.

[60] Goldman D, Smith JP (2011). The increasing value of education to health. Soc Sci Med (1982).

[61] Green MJ, Benzeval M (2013). The development of socioeconomic inequalities in anxiety and depression symptoms over the lifecourse. Soc Psychiatry Psychiatr Epidemiol.

[62] Mehregan N, Ghasemifar S, Sohrabi Vafa H, Rashid K (2016). The impact of economic and social conditions on mental health across provinces of Iran (1378-1391). Majlis Rahbord.

[63] Oh DH, Park JH, Lee HY, Kim SA, Choi BY, Nam JH (2015). Association between living arrangements and depressive symptoms among older women and men in South Korea. Soc Psychiatry Psychiatr Epidemiol.

[64] Erzen E, Çikrikci Ö (2018). The effect of loneliness on depression: a meta-analysis. Int J Soc Psychiatry.

[65] Kraav SL, Lehto SM, Junttila N, Ruusunen A, Kauhanen J, Hantunen S (2021). Depression and loneliness may have a direct connection without mediating factors. Nordic J Psychiatry.

[66] Tragantzopoulou P, Giannouli V (2021). Social isolation and loneliness in old age: Exploring their role in mental and physical health. Psychiatriki.

[67] Kawachi I, Berkman L, Berkman LF, Kawachi I (2000). Social cohesion, social capital, and health. Social Epidemiology.

[68] Singh L, Singh PK, Arokiasamy P (2016). Social network and mental health among older adults in Rural Uttar Pradesh, India: a cross-sectional study. J Cross Cult Gerontol.

[69] Lavela SL, Ather N (2010). Psychological health in older adult spousal caregivers of older adults. Chronic Illn.

[70] Gou Y, Wu N, Xia J, Liu Y, Yang H, Wang H (2022). Province- and individual-level influential factors of depression: multilevel cross-provinces comparison in China. Front Public Health.

